# Analgesia and Sedation an Essential Adjunct to Treatment

**Published:** 1895-12

**Authors:** John J. Sullivan

**Affiliations:** University of the City of New York; 266 West 38th Street, New York City


### ANALGESIA AND SEDATION AN ESSENTIAL ADJUNCT TO TREATMENT.

BY JOHN J. SULLIVAN, M. D., UNIVERSITY OF THE CITY OF NEW YORK.

On account of the frequency with which pneumonia, in late years, is accompanied with grippal symptoms, the treatment, to a great extent, has been modified or changed. The essential features in the result desired are a diminution of the pain and a lowering of the temperature. Opinions differ as to whether a reduction of the temperature influences the course of the disease, but a concensus of opinion is that *antipyretic* treatment is distinctly called for in the beginning and analgesic at all times, if needed to assuage suffering. *The antipyretic should be antikamnia, and the analgesic is supplied by codeine and antikamnia together.* This is given every three or four hours in tablets containing four and three-fourths grains antikamnia and one-fourth grain codeine throughout the period of congestion and consolidation. Where there is great restlessness, this will have a delightful effect.

In the nocturnal pains of syphilis, in the grinding pains which precede labor, and the uterine contractions which often lead to abortion, in tic douloureux, brachialgia, cardialgia, gastralgia, hepatalgia, nephralgia, and dysmenorrhœa, immediate relief is afforded by the use of this combination, and the relief is not merely temporary and palliative, but in very many cases, curative.

In the neuroses of the respiratory organs, great relief is afforded by the use of this combination. A paroxysm of asthma is often cut short by a full dose; hay fever or autumnal catarrh is benefited by its use.

In the harassing cough of phthisis, or in the pain of pleuritis, in the painful sensations accompanying bronchitis when the tubes are dry and irritable—as they usually are—the blending of codeine and antikamnia will not be found wanting in its action, but will give results that are gratifying to both the patient and the medical attendant. As a producer of sleep it will be found efficacious. This is doubly true when there is great nervous excitement.

In pulmonary diseases this combination is worthy of trial. It is a sedative to the respiratory centers in both acute and chronic disorders of the lungs. Cough, in the vast majority of cases, is promptly and lastingly decreased and often entirely suppressed. In diseases of the respiratory organs, pain and cough are the symptoms which especially call for something to relieve; this tablet does it, and in addition controls the violent movements accompanying the cough, which are so distressing.

This combination is the remedy for diabetes and is superior to any other in diminishing the quantity of sugar in the urine, and also in diminishing the quantity of urine itself in diabetis mellitus. The bulimia and polydipsia are lessened by its use, and probably the changes in the nervous system which accompany or are causative of the disease, are arrested or prevented. It also prevents waste; it controls restlessness; it relieves insomnia; it relieves distressing nervous symptoms. It relieves the craving of the stomach and lessens the frequency of the calls to urinate.

It is not claimed that the combination will cure diabetis mellitus, but there will be, in many cases, arrest of the disease, with prolonged periods of good health, and cure, in some cases.

*266 West 38th Street, New York City.*

---

A QUACK CONVICTED.—It is gratifying to announce that at last something is being done in enforcing the medical practice law. On December 4th, a verdict of guilty was rendered against one Stark, a so-called hypnotist and a very obvious quack, for practicing medicine without a license. The penalty, $5.00 fine and cost, was absurd, still it means that the authorities are at last waking up. We sincerely trust that the good work will be kept up until not one of these obscene birds is left.

